# Climate change and the loss of organic archaeological deposits in the Arctic

**DOI:** 10.1038/srep28690

**Published:** 2016-06-30

**Authors:** Jørgen Hollesen, Henning Matthiesen, Anders Bjørn Møller, Andreas Westergaard-Nielsen, Bo Elberling

**Affiliations:** 1 Department of Conservation and Natural Sciences, National Museum of Denmark, I.C. Modewegsvej, Brede, DK-2800 Lyngby, Denmark; 2 Center for Permafrost (CENPERM), Department of Geoscience and Natural Resource Management, University of Copenhagen, Øster Voldgade 10, DK-1350, Copenhagen K. Denmark; 3 Department of Agroecology, Aarhus University, Blichers Allé 20, DK-8830, Tjele, Denmark

## Abstract

The Arctic is warming twice as fast as the global average with overlooked consequences for the preservation of the rich cultural and environmental records that have been stored for millennia in archaeological deposits. In this article, we investigate the oxic degradation of different types of organic archaeological deposits located in different climatic zones in West and South Greenland. The rate of degradation is investigated based on measurements of O_2_ consumption, CO_2_ production and heat production at different temperatures and water contents. Overall, there is good consistency between the three methods. However, at one site the, O_2_ consumption is markedly higher than the CO_2_ production, highlighting the importance of combining several measures when assessing the vulnerability of organic deposits. The archaeological deposits are highly vulnerable to degradation regardless of age, depositional and environmental conditions. Degradation rates of the deposits are more sensitive to increasing temperatures than natural soils and the process is accompanied by a high microbial heat production that correlates significantly with their total carbon content. We conclude that organic archaeology in the Arctic is facing a critical challenge that requires international action.

Archaeological deposits in Arctic areas are known to hold extraordinary well-preserved organic archaeological materials, primarily due to the cold temperatures and in some cases lack of water or oxygen within the depositional environments^1^,^2^,^3^,^4^. In addition to the archaeological materials, the deposits are often rich in residues from animals, plants, insects, and pollen^5^,^6^ and are therefore increasingly being recognized as valuable paleoclimatic and paleoenvironmental archives^7^.

Climate change is having a great impact on the Arctic environment^8^. Increasing air temperatures cause longer periods of ground thawing and changes in precipitation affect the hydrology and the amounts of oxygen that diffuse into the ground with impact on the microbial decomposition of organic carbon^9^,^10^,^11^. The until now well-preserved archaeological deposits may thereby be exposed to accelerated degradation that destroys the integrity of stratified layers and leads to the loss of key elements such as archaeological wood, bone and ancient DNA. While the physical destruction due to e.g. coastal erosion has been studied to some extent^12^, very little is known about how climate change affects the microbial degradation of organic archaeological deposits and therefore the extent of this threat to our common heritage remains largely unknown. To our knowledge, the coupling between climate change and the degradation of organic archaeological deposits has only been studied at one site in the Arctic, i.e. the Qajaa kitchen midden in the Disco Bay area in Greenland. Results from Qajaa have shown that even a small increase in soil temperature may lead to substantial degradation of the buried remains^13^,^14^. Furthermore, the organic-rich archaeological deposits at Qajaa produce enough heat during decomposition to further increase soil temperatures and consequently accelerate the rate at which the organic matter is degraded^15^. So far it is unknown whether these results are site-specific or whether the many well-preserved organic archaeological deposits located in other Arctic areas are equally vulnerable.

In this study we assess the vulnerability of organic archaeological deposits at four contrasting sites located along the two main climatic gradients in West and South Greenland. The degradability of the archaeological deposits is investigated at different temperatures and water contents by measuring O_2_ consumption, CO_2_ production and micro calorimetric heat production in depth-specific samples from the study sites. As far as we know, this is the first time these three methods are used in combination to study archaeological materials and hence the results presented here also provide a unique methodological insight that may be useful whenever assessing the vulnerability of organic archaeological deposits.

## Results

### Study sites and environmental conditions

**Figure 1:**
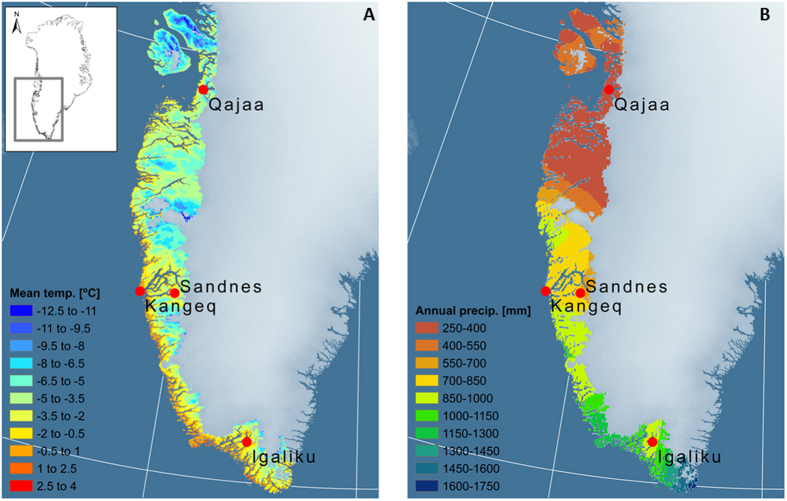
The four study sites are located in West and South Greenland. **(A)** Mean annual temperatures derived from the MODIS-based land surface temperature product MOD11A2^16^ and **(B)** accumulated annual precipitation from the ERA Interim reanalysis dataset^17^. Both datasets were analyzed for the period 2005–2014. Figure 1 is generated in ArcMap 10.2.2 (Environmental Systems Research Institute (ESRI) http://www.esri.com/software/arcgis), using the Layout View panel.

The four archaeological sites included in this study, Qajaa, Sandnes, Kangeq, and Igaliku, are among the most valuable sites in Greenland and all contain well-preserved organic materials (Fig. 1 and Supplementary Figs S1–7). The sites were chosen in order to represent different types of archaeological deposits that have been exposed to the different climatic conditions found in West and South Greenland. Qajaa consists of deposits from the three main Eskimoic cultures of Greenland: Saqqaq (2,500–800 BC), Dorset (300 BC–600 AD) and Thule (1,300 AD–present), Kangeq of deposits from the Thule culture (1,300 AD–present), Sandnes of deposits from the Norse farmers inhabiting the Western Settlement (985–1,350 AD) and Igaliku of deposits from the Norse farmers inhabiting the Eastern Settlement (985–1,450/1,500 AD).

**Figure 2:**
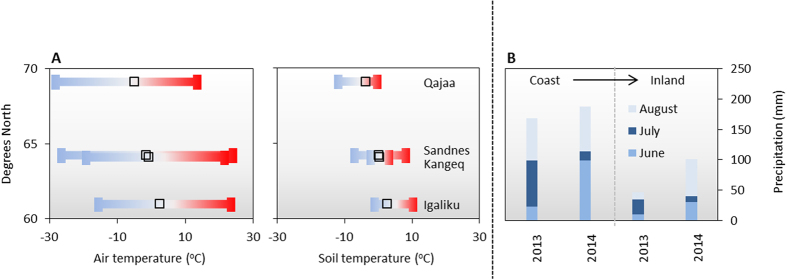
**(A)** The mean annual air temperatures (left) and soil temperatures in 50 cm depth (right) measured at the study sites from 15 August 2013 to 15 August 2014. The horizontal color bars show the variation from the minimum to the maximum mean daily temperature during this period. **(B)** Monthly precipitation rates during the summer periods of 2013 and 2014 at Kangeq/Nuuk (left) and Sandnes (right). Precipitation rates are not measured at Kangeq instead values from the nearby meteorological station in Nuuk are used (located 20 km to the East). These data are provided by the Danish Meteorological Institute^38^.

The study sites are located along a North/South gradient with respect to air temperatures and soil temperatures (Fig. 2A) and a West/East gradient with respect to precipitation (Fig. 2B). Permafrost was found at the northernmost site Qajaa (continuous permafrost) and at the inland site of Sandnes (sporadic permafrost). The soil water contents varied substantially within and between the sites with the inland site of Sandnes being relative dry compared to the three other coastal sites (Supplementary Fig. S8).

The representativeness of the sites in terms of climatic conditions was evaluated against mean annual temperatures derived from the MODIS-based land surface temperature product MOD11A2^16^ as well as accumulated annual precipitation from the ERA Interim reanalysis dataset^17^. Both datasets were analyzed for the period 2005–2014 (Fig. 1 and Supplementary Table S1). The mean annual temperatures at the four study sites represented about 40% of the total ice-free land area in West and South Greenland and 65% of the ice-free land below 100 m a.s.l. where the majority of archeological sites are located (Supplementary Table S2). In terms of precipitation, the four study sites represented roughly 70% of the precipitation regimes found in the ice-free land areas in West and South Greenland (Supplementary Table S2).

### Degradability of archaeological deposits

**Figure 3:**
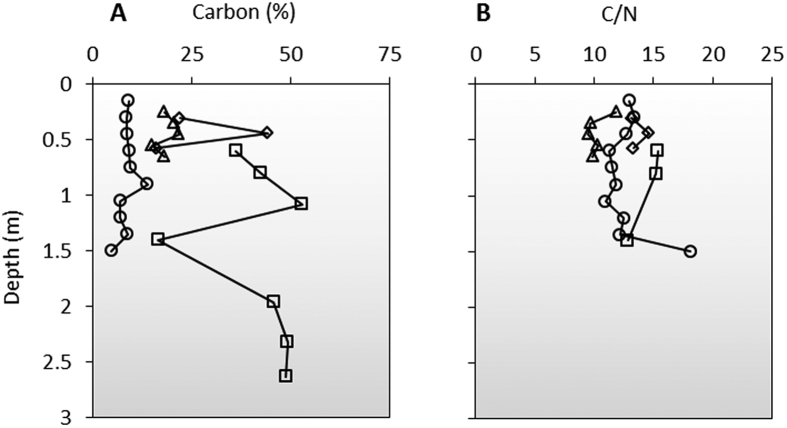
Vertical profiles of **(A)** carbon content and **(B)** C/N ratios at Qajaa (squares), Sandnes (circles), Kangeq (triangles), and Igaliku (diamonds).

Soil bulk samples and volume specific samples (100 cm^3^) were collected at each site for laboratory experiments. The carbon content (TOC) varied considerably both within and across locations (Fig. 3A) with the samples from Sandnes containing least organic C (8.5 ± 2.3% weight to dry weight) (mean  ±  SD; n = 10) and the samples from Qajaa the most (42 ± 12%) (mean  ±  SD; n = 7). The C/N relationship varied between 10 and 18 in all the samples (Fig. 3B). The pyrite content was measured in selected samples and was <0.02% at Qajaa, 0.05–0.08% at Sandnes, 0.22% at Kangeq and 0.37% at Igaliku.

**Figure 4:**
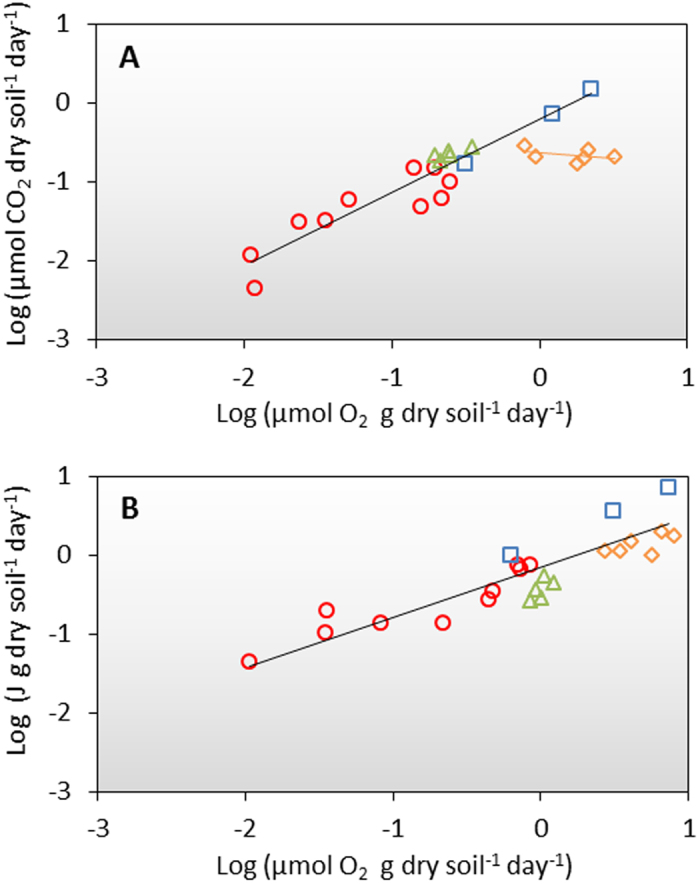
**(A)** Linear correlation between logarithmic transformed O_2_ consumption rates and CO_2_ production rates measured at 5 °C, **(B)** Linear correlation between logarithmic transformed O_2_ consumption rates and heat production rates at 16 °C. Measurements were made on samples from Qajaa (squares), Sandnes (circles), Kangeq (triangles), and Igaliku (diamonds). The solid lines in the upper figure represent the linear fits for all points excluding Igaliku (black) and for Igaliku alone (orange). The solid black line in the bottom figure represents the linear fit for all points.

The rate of degradation was investigated in 24 depth-specific samples from the four sites (Supplementary Table S3). This was done by measuring O_2_ consumption, CO_2_ production and micro calorimetric heat production on triplicates of each sample (Fig. 4 and Supplementary Figs S9 and S10). At 5 °C, the O_2_ consumption rates varied between 0.01 and 3.21 μmol O_2_ g dry soil^−1^ day^−1^ (mean = 0.71 ± 0.88 μmol O_2_ g dry soil^−1^ day^−1^) (mean  ±  SD; n = 24) and the CO_2_ production rates between 0.00 and 1.50 μmol CO_2_ g dry soil^−1^ day^−1^ (mean = 0.23 ± 0.30 μmol CO_2_ g dry soil^−1^ day^−1^) (mean  ±  SD; n = 24). The rates increased markedly with depth at Qajaa and Sandnes, whereas the depth-specific variation was limited at Kangeq and Igaliku (Supplementary Fig. S9). For Qajaa, Kangeq and Sandnes, the O_2_ consumption and CO_2_ production rates were significantly positively correlated (linear regression on logarithmic transformed data: r = 0.94, p < 0.01, n = 18) with a slope (respiratory quotient) of 0.93 ± 0.08 mol CO_2 _mol^−1^ O_2_ (±SD of slope; n = 18). For Igaliku, the correlation between the O_2_ consumption and CO_2_ production was poor (linear regression on logarithmic transformed data: r = −0.32, p > 0.05, n = 6), and the respiratory quotient of the samples was very low (−0.64 ± 0.05 mol CO_2 _mol^−1^ O_2_) (± SD of slope; n = 6).

The O_2_ consumption and heat production rates were measured at 16 °C and compared to each other (Fig. 4B). The O_2_ consumption rates varied between 0.01 and 8.09 μmol O_2_ g dry soil^−1^ day^−1^ (mean = 2.10 ± 2.50 μmol O_2_ g dry soil^−1^ day^−1^) (mean ± SD; n = 24) and the heat production between 0.0 and 7.08 J g dry soil^−1^ day^−1^ (mean = 1.05 ± 1.51 J g dry soil^−1^ day^−1^) (mean ± SD; n = 24) (Supplementary Fig. S10). Overall, the heat production was significantly positively correlated with the O_2_ consumption (linear regression on logarithmic transformed data: r = 0.89, p < 0.01, n = 24) (Fig. 4B). The ratio between the heat production and oxygen consumption was 587 ± 66 kJ mol^−1^ O_2_ (slope of linear correlation curve ± SD of slope), with the samples from Qajaa producing more heat than the samples from Igaliku (Fig. 4B).

**Figure 5:**
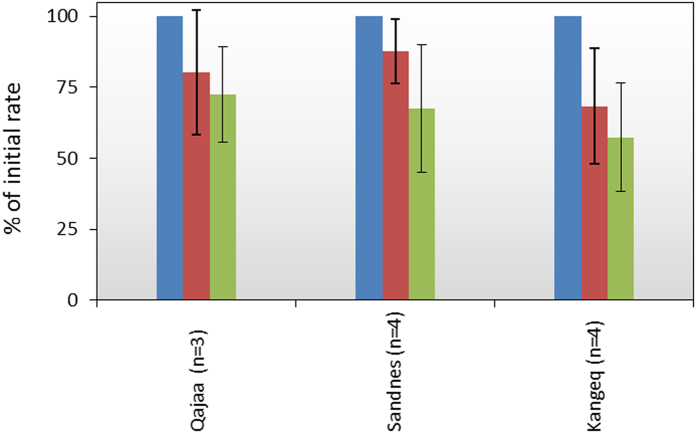
Decrease in the O_2_ consumption rate with time in samples taken from Qajaa, Sandnes, and Kangeq. Blue bars show the initial rate, red bars the rate after 6 months and green bars the rate after 14 months. The rates are normalized in relation to the initial rate. The n-values represent the number of different archaeological layers investigated. Error bars show ± 1 standard deviation.

The O_2_ consumption measurements were repeated after 6 and 14 months in selected samples from Qajaa, Sandnes and Kangeq to investigate the long-term degradation of the archaeological deposits. During this 14 month period, the O_2_ consumption rates in average decreased to 61 ± 23% (mean ± SD; n = 11) of the initial rate (Fig. 5). This average represents significant decreases in O_2_ consumption rates to 67% at Sandnes (one way ANOVA: p = 0.03, n = 4) and to 57% at Kangeq (one way ANOVA: p = 0.01, n = 4) whereas the decrease at Qajaa to 72% of the initial rate is not significant (one way ANOVA, p = 0.17, n = 3).

### Vulnerability to climate change

The temperature dependence of the O_2_ consumption rates was investigated in the temperature interval from 0.5 to 16 °C in order to evaluate the vulnerability of the archaeological deposits to changes in temperature. A significant exponential increase in reactivity with increasing temperature was observed (Supplementary Fig. S11; Supplementary Table S4) which is consistent with studies of natural soils^18^. The increase in activity per 10 °C temperature change (the Q_10_ value) varied between 1.8 and 5.1 with mean values of 2.5 ± 0.7 at Qajaa (mean ± SD; n = 3), 3.4 ± 0.5 at Sandnes (mean ± SD; n = 5), 3.9 ± 1.1 at Kangeq (mean ± SD; n = 5), and 3.2 ± 1.0 at Igaliku (mean ± SD; n = 3).

The importance of soil water on the degradation rates was investigated (at 16 °C) by measuring the O_2_ consumption in selected samples from Sandnes, Kangeq and Igaliku (Supplementary Fig. S12). In this experiment, sterilized deionized water was first added to mimic saturated conditions where the oxygen supply is limited, and after the first round of measurements, the samples were freely drained and exposed to air for 24 h to mimic oxic conditions before repeating the measurements. Results showed an oxygen consumption in the wet samples between 1.3 to 2.6 μmol O_2_ g dry soil^−1^ day^−1^ (mean = 1.8 μmol O_2_ g dry soil^−1^ day^−1^) (mean ± SD; n = 7), but upon drainage, the average reactivity was 2.5 times higher. Furthermore, adding water to the very dry samples from the deposits at Sandnes increased the reactivity 110 times.

## Discussion

We included 24 depth-specific samples taken from four contrasting types of archaeological deposits that contained well-preserved organic materials from the main cultures who have lived in West and South Greenland during the past 4,000 years. In line with previous studies^19^,^20^, the collected climate data reveal a North/South (cold/warm) gradient in air temperatures and soil temperatures (Fig. 2A) and a West/East (wet/dry) gradient in precipitation (Fig. 2B). Comparison with regional climate data shows that the mean annual air temperatures and precipitation rates at the four study sites represent 60–70% of the ice-free coastal areas in West and South Greenland (Fig. 1 and Supplementary Table S1, S2). According to the official Greenlandic heritage data base, several thousand archaeological sites are registered in this part of Greenland (Supplementary Fig. S13) and we therefore expect that a large number of archaeological sites are exposed to environmental conditions similar to our study sites.

A combination of O_2_ consumption, CO_2_ production and heat production measurements was used to investigate the degradability of the archaeological deposits. The measured degradation rates confirm that the archaeological deposits are highly reactive with rates that are in the same order of magnitude as what has been reported from organic permafrost soils in Greenland^15^. For three out of four sites, we found a good correlation between CO_2_ production and O_2_ consumption with a ratio of approximately 0.93 mol CO_2 _mol^−1^ O_2_. This is within the normal range found for the decomposition of soil organic matter and indicates that O_2_ is mainly consumed by aerobic mineralization of SOC with a low oxidation level (low O-content)^21^ (Fig. 4A). For the last site (Igaliku), the O_2_ consumption was 3–15 times higher than the CO_2_ production, indicating that processes other than SOC oxidation are taking place. Measurements of the pyrite (FeS_2_) content showed that the deposits at Igaliku contain pyrite (0.37%), indicating that O_2_ could also be consumed in reaction with pyrite. As both pyrite and SOC produces heat when oxidized this also explains why the O_2_ consumption and heat production was correlated at all four sites. This highlights the advantage of combining several methods when assessing the degradation of organic materials as e.g. carbonate dissolution may produce CO_2_ without consuming O_2_ and pyrite oxidation may consume O_2_ without reflecting the degradation of organic artifacts directly.

For Qajaa, Sandnes and Kangeq, the rates of oxygen consumption, CO_2_ production and heat production were all significantly correlated to the carbon content (linear regression on logarithmic transformed data: O_2_ vs C: r = 0.73, p < 0.01, n = 18; CO_2_ vs C: r = 0.76, p < 0.01, n = 18; heat production vs C: r = 0.73, p < 0.01, n = 18) (Supplementary Figs S14 and S15). Thereby, the organic carbon found at the three sites is almost equally reactive regardless of the sites being very different in age and being exposed to different depositional/environmental conditions. In addition, the measured C/N ratios (ranging from 10 to 18, Fig. 3B) were all within the range of what is considered optimal for C decomposition^22^. This verifies the hypothesis that the degradation so far has been limited by other parameters such as lack of water and/or oxygen, in combination with the low temperatures, and thus further suggests that Arctic archaeological deposits are highly degradable and vulnerable to environmental change.

With Q_10_ values between 1.8 and 5.1 (mean = 3.3 ± 0.9), the archaeological deposits are more sensitive to temperature change than Arctic organic soils in general^23^. In 2100, the mean annual air temperature in Western Greenland is expected to be 4.0–7.0 °C warmer than the 1961–1990 mean^24^. This could in average increase in the degradation rate by 62–132%; but for the layers with Q_10_ values above 5 the increase could be as high as 200%. This will be highly dependent on the future water balance and thus, on whether O_2_ will be available in the deposits. Climate- and atmospheric models generally suggest a future increase in precipitation in the Arctic^25^,^26^. However, a likely future increase in evapotranspiration and runoff is also reported which could result in lower soil moisture content especially in the southern parts of the region^8^. Our measurements show that draining of saturated samples increased the oxygen consumption rates. For sites that are currently wet, drier conditions may lead to increased oxygen access that will accelerate the degradation. On the other hand, wetter conditions may help to keep the archaeological deposits water saturated and reduce the microbial activity. The measurements also show a 110 times increase in reactivity when water is added to the very dry samples from the inland site of Sandnes. This suggests that at sites that are currently very dry, increasing precipitation could improve the conditions for microbial activity and accelerate the rate of degradation.

Previously, it has been shown that the impact of climate change on the archaeological deposits at Qajaa can be accelerated by microbial heat production^15^. The results obtained here further highlight the fact that microbial heat production is seen across different types of sites and is positively correlated with the organic C content of the deposits (= 0.73, p < 0.01, n = 18. The overall warming effect from heat production is expected to be most pronounced in deposit with a very high organic C content, where the degradation is not limited by lack of oxygen or water. Furthermore, in accordance with studies from Arctic mine waste rock dumps^27^,^28^, the thickness and volume of the archaeological deposits are important for the overall “internal warming effect” of microbial heat production.

We investigated the long-term degradation of the archaeological deposits and found that oxygen consumption rates in average decreased to 61 ± 23% of the initial rate after 14 months (Fig. 5). This is in good agreement with studies of natural soils showing that the most reactive organic material decomposes first and, with time, the remaining material becomes decreasingly reactive^9^,^29^. This is important when quantifying the long term loss of organic material and the corresponding emissions of CO_2_ to the atmosphere. In relation to archaeological deposits, the mineralization over the first few years is considered more critical than in natural soils, as the initial degradation can cause irreversible damage to the quality and integrity of the deposits.

In this study, we have focused on the microbial degradation of organic deposits that contain important residues of human subsistence activities and in which the different types of artefacts are embedded. Previously, it has been have shown that archaeological wood is equally vulnerable when exposed to O_2_ and higher temperatures^14^. We therefore expect the loss of organic deposits to be closely linked to a loss in quality of other buried organic artefacts. Furthermore, the loss of organic material may be accompanied by soil settling and erosion processes that accelerate the destruction of their cultural and scientific value.

## Conclusion

We have shown that archaeological deposits from four contrasting archaeological sites located in different climatic zones in West and South Greenland all are highly reactive and vulnerable to increasing soil temperatures and changes in the water balance. The results provide strong evidence that Arctic organic archaeological deposits, regardless of age and geographical location, are particularly vulnerable to degradation under the climatic changes predicted by global climate models.

For most samples, we found a good correlation between CO_2_ production and O_2_ consumption, confirming that most of the O_2_ is consumed by reacting with organic carbon in the deposits. However, for one of the sites, the correlation was poor, indicating other ongoing oxygen consuming processes. This highlights the importance of combining several methods when assessing the degradation of organic materials, especially when quantifying their long term loss.

The organic carbon found in the archaeological deposits has a significant heat production potential that may be enough to warm the deposit and thereby accelerate the degradation more than can be attributed to the direct impact of climate change alone. The effect of this heat production is highly dependent on the total carbon content, the thickness of the deposits and the local environmental conditions.

It is difficult to discover, quantify and predict ongoing microbial or chemical degradation of buried deposits and, as a result, sites that are suddenly undergoing rapid degradation can be easily overlooked. The investigations presented here should be expanded to include more sites and other types of organic archaeological materials. This would provide the fundamental knowledge needed to develop regional impact models that can be used to pinpoint areas most vulnerable to microbial degradation. Such information will make it possible to prioritize and optimize future archaeological investigations and save important records of the past before they are lost.

## Methods

### Sites, monitoring and sampling

The northern site of Qajaa is located 18 km south-east of Ilulissat at the southern side of the Ilulissat Icefjord (69.127602° N, 50.702076° E). The midden at the site covers an area of approximately 2900 m^2^ and is up to 3 m thick (Supplementary Fig. S1). The midden contains remains of the three main Eskimoic cultures of Greenland: The Saqqaq, Dorset and Thule cultures, and the Saqqaq remains are considered some of the best preserved in Greenland^30^ (Supplementary Fig. S2). The midden is permanently frozen with an active layer (top layer of soil that thaws during the summer) of approximately 0.5 m. The site was visited in 2009, 2010, 2011, 2012 and 2014. Part of the field work carried out at the site has been published^13^,^14^,^15^,^24^. In 2009, a permafrost core was extracted from the site, after which temperature probes (Campbell Scientific T107 temperature probes) were installed at 7, 16, 32, 50, 120, 170, 220, 270 and 320 cm depth. Soil water Theta Probes (ML2x, Delta-T Devices Ltd, Cambridge, UK) were installed in the active layer at the depths 7, 16, 20 and 32 cm. In 2010, a meteorological station was installed, monitoring the air temperature (Campbell Scientific CS215 temperature probe) (Supplementary Fig. S2). Soil bulk samples and volume specific samples (100 cm^3^) from an exposed, north-facing profile (Profile A) were included in this study.

The coastal site of Kangeq is located in the archipelago outside Nuuk (64.106667° N, 52.051033° E). The midden consists of several patches around a small inlet and is up to 2.5 m thick (Supplementary Fig. S3). Most of the midden was accumulated by the Thule culture, but layers from the Saqqaq and Dorset cultures are also present^31^,^32^ (Supplementary Fig. S4). The midden is not permanently frozen, but still contains well-preserved organic artefacts including wood, mollusk shells and feathers. The site was visited in 2012, 2013 and 2014. In 2012, a pit was excavated vertically into the midden. Bulk samples were collected from depths of 25, 35, 45, 55 and 65 cm, and volume specific samples were collected from depths of 20, 30 and 50 cm (Supplementary Fig. S4). Temperature probes (Campbell Scientific T107 temperature probes) were installed at depths of 0, 10, 20, 30, 40, 60, 80 and 100 cm. An additional probe was installed above the surface in order to monitor the air temperature. Hobo^®^ (S-SMx-M005) soil water sensors connected to a Hobo^®^ Micro Station were installed at depths of 10, 20, 30 and 40 cm.

The inland site of Sandnes is located on an alluvial fan of sand and gravel which forms a cape at the head of the Ameralik Fjord approx. 100 km west of Nuuk (64.243400° N, 50.175167° W). The midden is located on the eastern shoreline of the cape, is up to 1.5 m thick and covers an estimated area of 1200 m^2^ (Supplementary Figs S5 and 6). The site, also known as Kilaarsarfik, was occupied by the Norse in medieval times. Sporadic permafrost has been observed at the site and most of the artefacts in the midden are animal bones but also well-preserved wood and animal manure has been found. The site was visited in 2012, 2013 and 2014. In 2012, a south-east facing profile from a previous excavation was cleaned (Supplementary Fig. S6). Bulk samples were collected from depths of 15, 30, 45, 60, 75, 90, 105, 120, 135 and 150 cm, and a total of 16 volume specific samples were collected at 10 cm intervals. Temperature probes (Campbell Scientific T107 temperature probes) were installed at depths of 0, 25, 50, 75, 100, 125 and 150 cm. Hobo^®^ (S-SMx-M005) soil water sensors connected to a Hobo^®^ Micro Station were installed at 25, 50, 75 and 125 cm depth. A meteorological station was installed approximately 100 m north of the midden, 8 m.a.s.l (Supplementary Fig. S6). The station was equipped to monitor the air temperature and relative humidity (MP100A Temperature & Relative Humidity probe) and precipitation (52202 Tipping Bucket Rain Gauge).

The southern site of Igaliku is located on a gentle east-facing slope at the head of the Igaliku Fjord (60.986466° N, 45.420535° W) (Supplementary Fig. S7). The culture layer investigated in this study is located underneath the peat of a meadow. It is up to 0.5 m thick, covers an area of up to 90 by 80 m. This Norse site has been identified as the medieval bishop’s seat of Gardar, and the culture layer contains a large number of well-preserved artefacts including worked wood and animal bones and has been characterized as a plaggen layer formed as a result of manuring^5^,^6^. The soil at the site is not permanently frozen. Igaliku was visited in 2013 and 2014. In 2013, two pits were excavated and volume specific samples were collected from depths of 10, 20, 30, 40, 50 and 60 bulk samples were collected from the top, middle and bottom of the cultural layer in the south-west corner of each pit. Temperature probes (Campbell Scientific T107 temperature probes) were installed at depths of 0, 10, 20, 30, 40 and 60 cm. An additional probe was installed above the surface in order to monitor the air temperature (Fig. S7). Hobo^®^ (S-SMx-M005) soil water sensors connected to a Hobo^®^ Micro Station were installed at depths of 5; 40 and 60 cm.

### Representativeness of sites

We evaluated the representativeness of the four study sites regarding air temperature and precipitation for the ice-free part of West and South Greenland (Fig. 1 and Supplementary Table S1). The mean annual temperature was evaluated using land surface temperatures (LST) derived from the MODIS sensor onboard the Terra satellite. LST from this sensor has shown to be closely linked with air temperatures in the Arctic^33^ , hence at 1 km ground resolution this product allows for detailed temperature mapping of the fjord systems dominating West and South Greenland. Annual mean LST from the period 2005–2014 was computed based on the 8-day product MOD11A2 and converted into air temperature 2 m above surface using a linear model derived from an ordinary least squares regression between air temperatures measured at the four study sites (2 m above terrain) and MODIS LST (0.8845 * LST + 1.626; r = 0.91; p < 0.001).

No similar high resolution precipitation products exist for the Arctic. Instead, we analyzed precipitation using the ERA Interim dataset at 0.125 degrees ground resolution, which we re-sampled to 0.05 degrees (approx. 5.5 km ground resolution, using bilinear interpolation) to allow masking of glaciers, open water, etc. The performance of this dataset in the Arctic has previously been documented^34^. Mean accumulated annual precipitation was then derived from two daily analyses in the period 2005–2014.

The MODIS and ERA Interim datasets were finally masked to cover only the terrestrial ice-free parts of West Greenland from 60 to 72^o^ N. The temperature and precipitation data was subsequently grouped into equally spaced groups, resulting in the classes reported in Supplementary Table S1. We used relative small intervals (1.5 °C and 150 mm year^−1^) compared to other studies^35^ in order to avoid the grouping of highly different climatic regimes. The spatial representativeness of the four research stations could then be quantified based on the specific air temperature and precipitation class covering the individual sites (Supplementary Table S2).

Lastly, the temperature dataset was re-analyzed using only elevations below 100 m a.s.l. based on the ASTER Global DEM^36^ and the same class intervals to allow comparison with low-lying areas only. Due to a significantly lower resolution of the ERA Interim reanalysis dataset, it was not possible to account for altitude in the precipitation analysis.

### Soil-physical and chemical properties

The total C content was measured on each bulk sample using a TruSpec^®^ CN Elemental Determinator (LECO Corporation) (Kangeq and Sandnes) and on a CS-500 Carbon/Sulfur Determinator (Eltra GmbH, Germany) (Qajaa and Igaliku). The N content was measured by means of a TruSpec^®^ CN Elemental Determinator (LECO Corporation). The pyrite content was measured in selected samples by stepwise extraction by first boiling the sample in 20% hydrochloric acid for 1 hour to remove all non-pyrite associated iron and sulphide. The extraction was followed by boiling of the washed sediment in concentrated 15% nitric acid for 1 hour releasing pyrite-associated iron and sulphide to solution^37^. Pyrite-associated iron in the acid solution was determined by atomic absorption spectroscopy (AAS).

### Incubation experiments

The collected soil bulk samples were homogenized manually (stones, bones, and wooden fragments were removed) and all the replicates used for the measurements of O_2_ consumption, CO_2_ production, and heat production were extracted. The samples, including the temperatures at which they were incubated, are listed in Supplementary Table S3. O_2_ consumption was measured on triplicates of each bulk sample by means of SDR SensorDish^®^ readers (PreSens Precision Sensing GmbH, Regensburg, Germany). The replicates were placed in vials, which were sealed by a disc of transparent commercial oxygen barrier film (EscalTM), a silicone gasket and a screw cap with aperture. Oxygen sensor foil (SF-PSt5-1223-01, PreSens) was glued to the inside of the oxygen barrier film, and the OxoDish^®^ readers were placed on top of the vials. At *in situ* water content, the O_2_ consumption was measured at 0.5, 5, 10 and 16 °C. In addition the importance of soil water on the degradation, rates were investigated (at 16 °C) by measuring the O_2_ consumption in selected samples from Sandnes, Kangeq and Igaliku. In this experiment, sterilized deionized water was first added to mimic saturated conditions, and after the first round of measurements, the samples were freely drained and exposed to air for 24 h to mimic oxic conditions before repeating the measurements.

The CO_2_ production was measured on triplicates from each bulk sample (with *in-situ* water contents) at 5 °C. This was done in a setup with an LI-840 CO_2_/H_2_O Gas analyzer (LICOR^®^ Biosciences) connected to a pump, two valves and a vial with a butyl septum via rubber tubing and two hypodermic needles. Gas samples with known volumes were injected into the analytical system through the septum, and their relative CO_2_ concentration was calculated as:
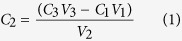
*C*_*1*_*: Relative CO*_*2*_ *concentration in the system before the sample is injected (ppm). V*_*1*_*: Volume of the system (ml). C*_*2*_*: Relative CO*_*2*_ *concentration of the sample (ppm). V*_*2*_*: Volume of the sample (ml). C*_*3*_*: Relative CO*_*2*_ *concentration in the system after the sample is injected (ppm). V*_*3*_*: Volume of the system and the gas sample (ml).*

The measurements were calibrated with certified standard gases (MikroLab, Aarhus, Denmark).

The heat production was measured calorimetrically on triplicates from each bulk sample on a Thermal Activity Monitor (TAM) 2277 (TA Instruments Inc.). During the measurements, the replicates were kept in stainless steel ampoules (4 ml twin, type 2277-201). Direct measurements of heat production rates are difficult to perform at temperatures lower than 15–16 °C due to the risk of condensation within the equipment. Therefore, the heat production was measured at 16 °C.

## Additional Information

**How to cite this article**: Hollesen, J. *et al*. Climate change and the loss of organic archaeological deposits in the Arctic. *Sci. Rep.* **6**, 28690; doi: 10.1038/srep28690 (2016).

## Supplementary Material

Supplementary Information
