# Supplementary material for: Climate change and the loss of organic archaeological deposits in the Arctic
Source: Sci Rep. 2016 Jun 30;6:28690. doi: 10.1038/srep28690 (PMC4928077; doi:10.1038/srep28690)
Supplement: Supplementary Information [file srep28690-s1.pdf]

Supplementary information:

Climate change and the loss of organic archaeological deposit in the Arctic

Jørgen Hollesen[1,2]†, Henning Matthiesen[1], Anders Bjørn Møller[3], Andreas Westergaard-Nielsen[2] and Bo Elberling[2]

[1]Department of Conservation and Natural Sciences, National Museum of Denmark, I.C. Modewegsvej, Brede, DK-2800 Lyngby, Denmark

[2]Center for Permafrost (CENPERM), Department of Geoscience and Natural Resource Management, University of Copenhagen, Øster Voldgade 10, DK-1350, Copenhagen K., Denmark

[3]Department of Agroecology, Aarhus University, Blichers Allé 20, DK-8830, Tjele, Denmark

†Corresponding author: e-mail joergen.hollesen@natmus.dk

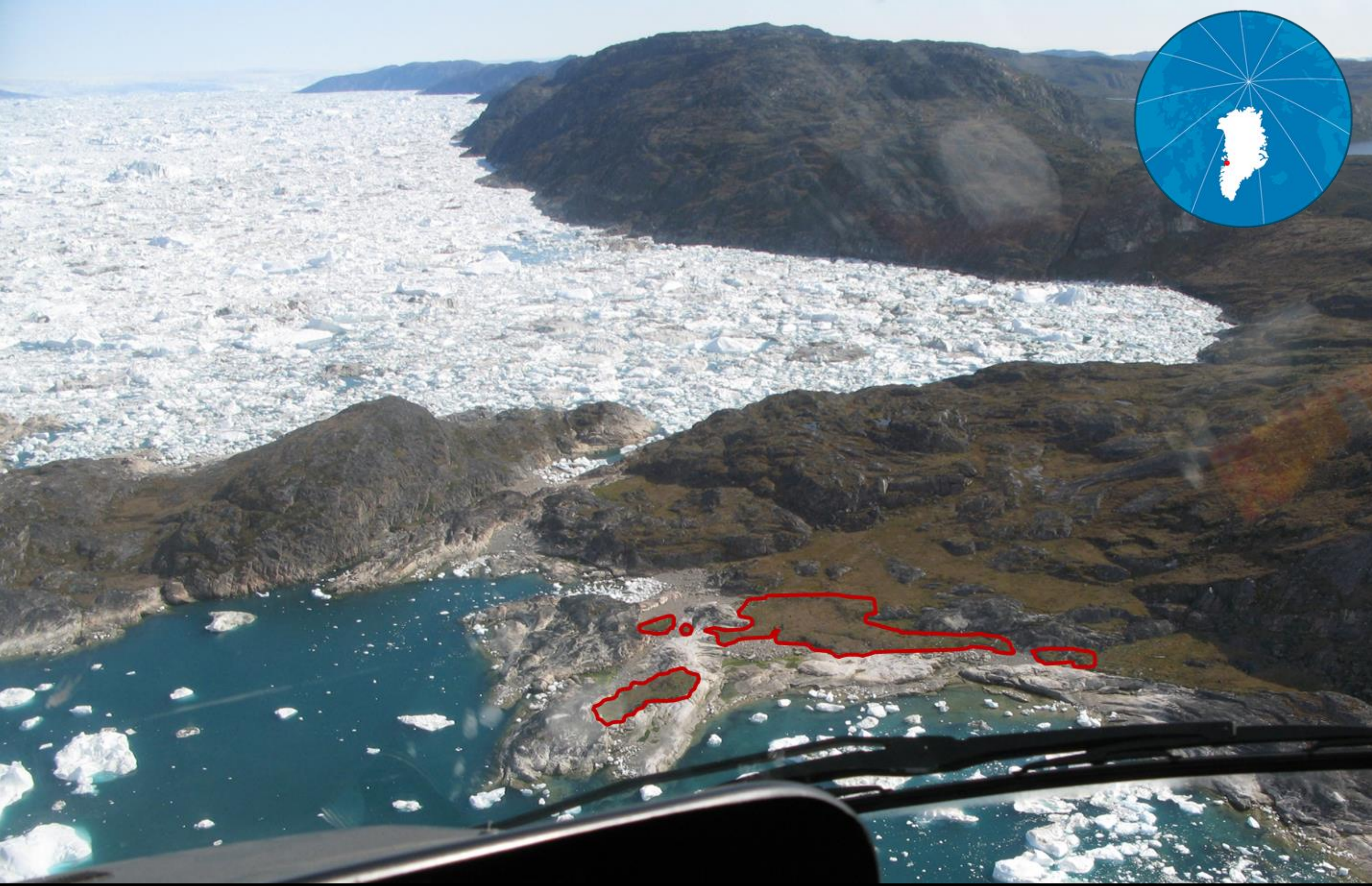

Supplementary figure S1: The midden at Qajaa (outline marked with red) is located on a small cape on the southern side of the Ilulissat Ice Fjord. Seen from the west. (Photo: Bjarne Grønnow, National Museum of Denmark; Map: Kent Pørksen, University of Copenhagen).

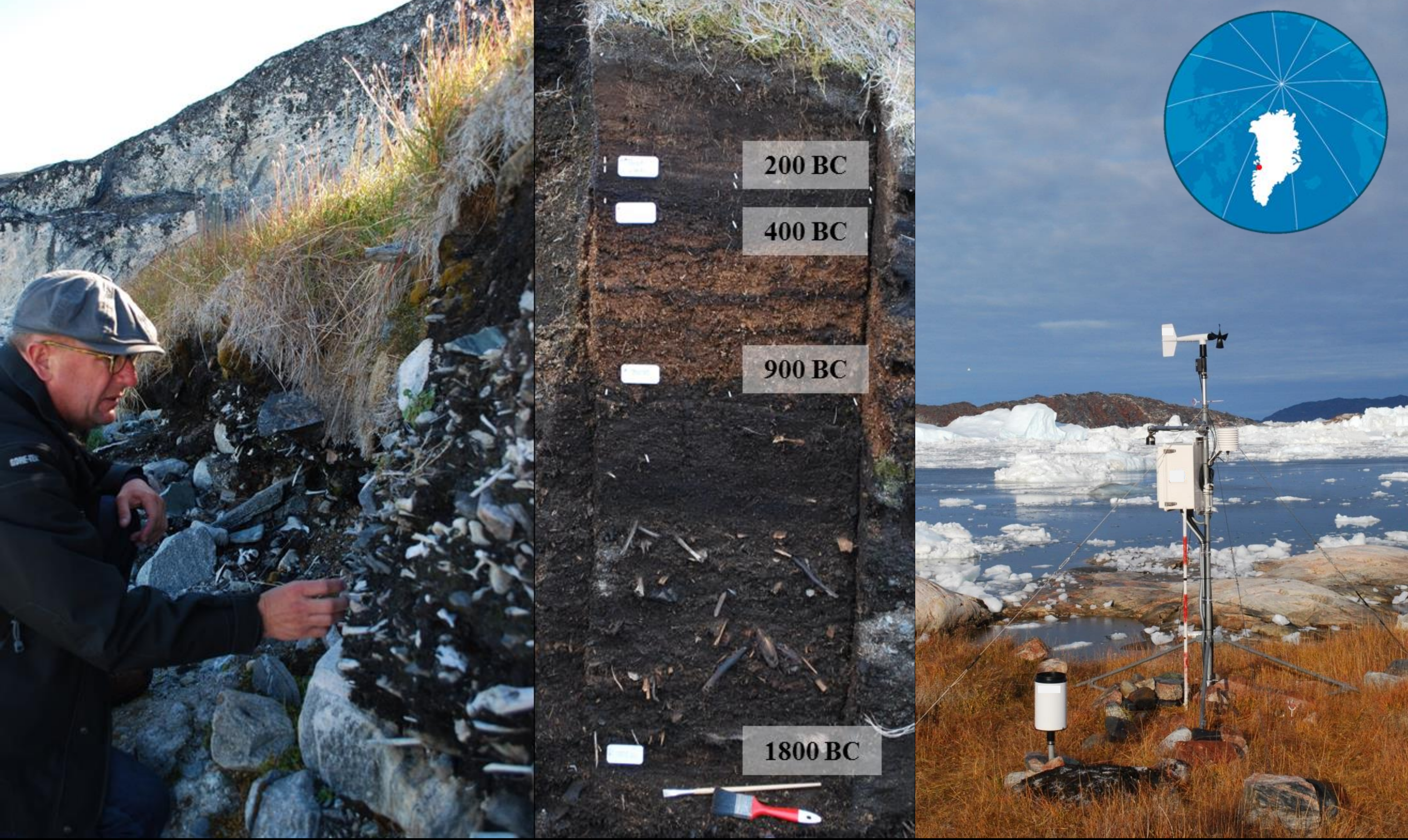

Supplementary figure S2: The midden at Qajaa is up to 3 m thick and contains remains of the the Saqqaq, Dorset and Thule cultures. In 2009 a permafrost core was extracted from the site, after which monitoring equipment was installed at various depths. The equipment was connected to a weather station with a datalogger (right picture). (Photo: Jesper Stub Johnsen, National Museum of Denmark (Left and middle) and Jørgen Hollesen, National Museum of Denmark (Right) ; Map: Kent Pørksen, University of Copenhagen).

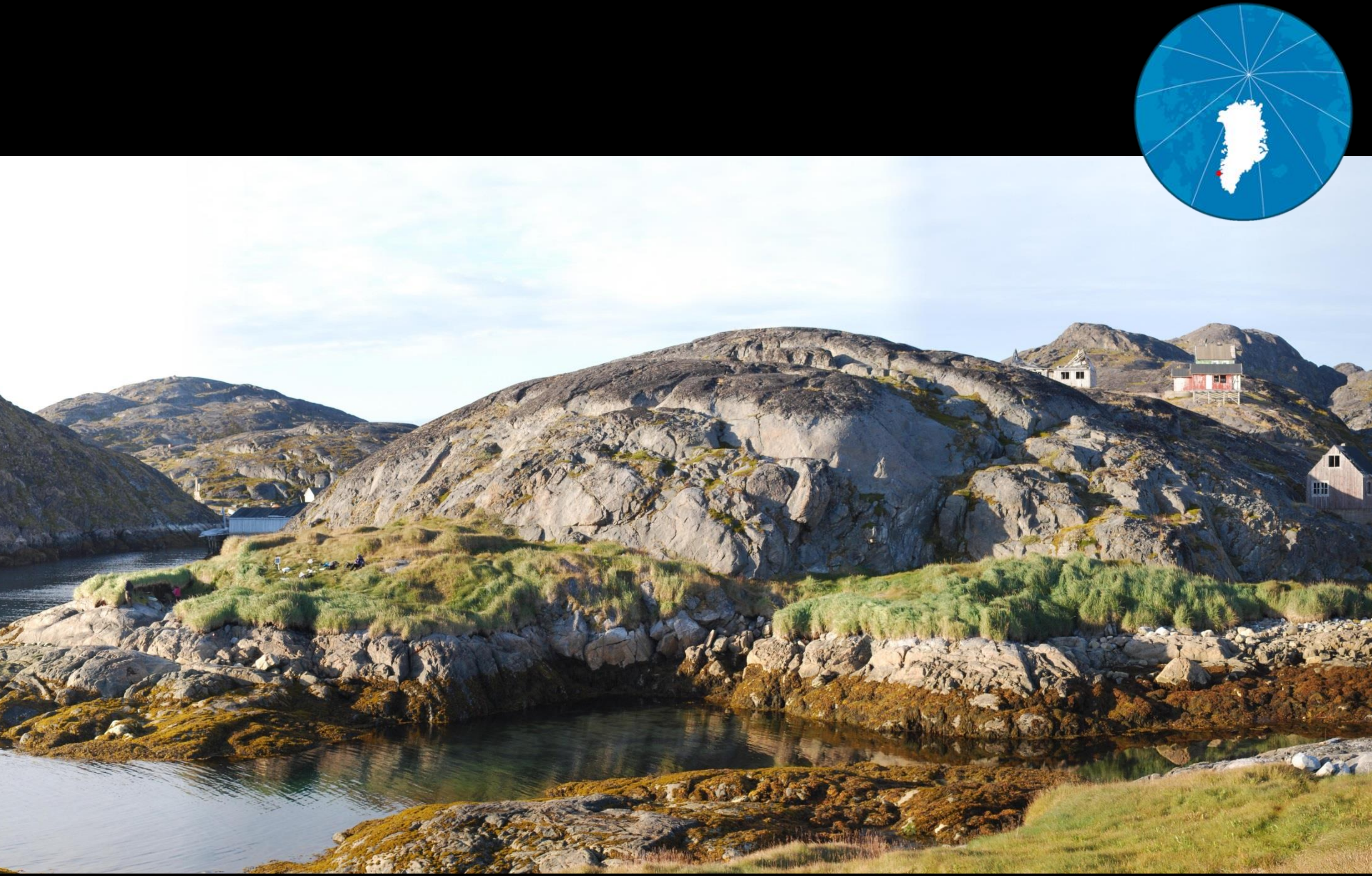

Supplementary figure S3: The midden at Kangeq is located around a small inlet in the archipelago outside of Nuuk. Seen from the South. (Photo: Jørgen Hollesen, National Museum of Denmark; Map: Kent Pørksen, University of Copenhagen).

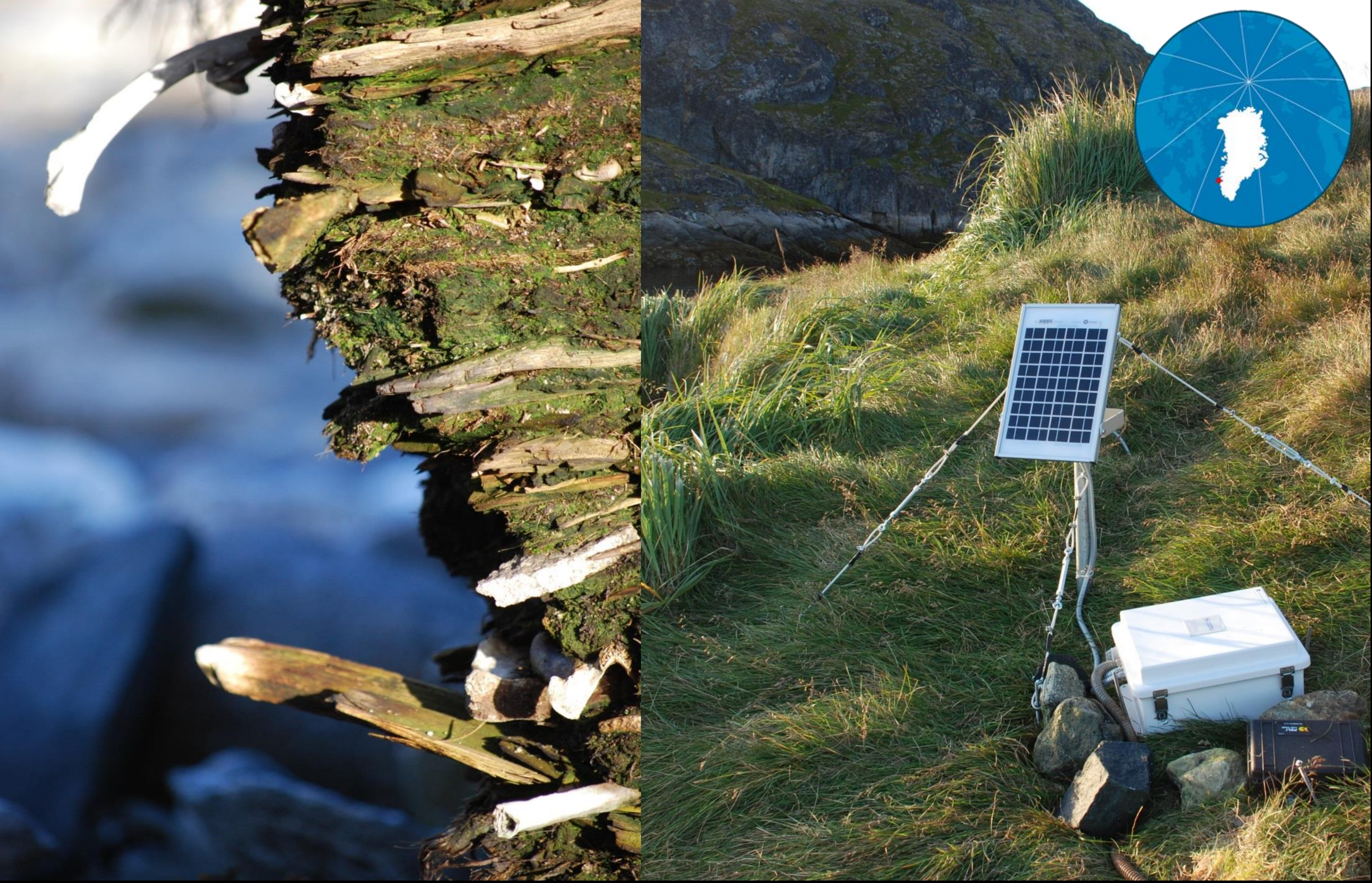

Supplementary figure S4: The midden at Kangeq contains well-preserved organic artefacts including wood, mollusk shells and feathers. Most of the midden was accumulated by the Thule culture (1,300AD–present) , but layers from the Saqqaq (2,500–800 BC) and Dorset (300BC–600AD) cultures are also present . In 2012 a pit was excavated vertically into the midden and temperature probes and soil water sensors were installed and connected to a datalogger (Right). (Photo: Jørgen Hollesen, National Museum of Denmark; Map: Kent Pørksen, University of Copenhagen).

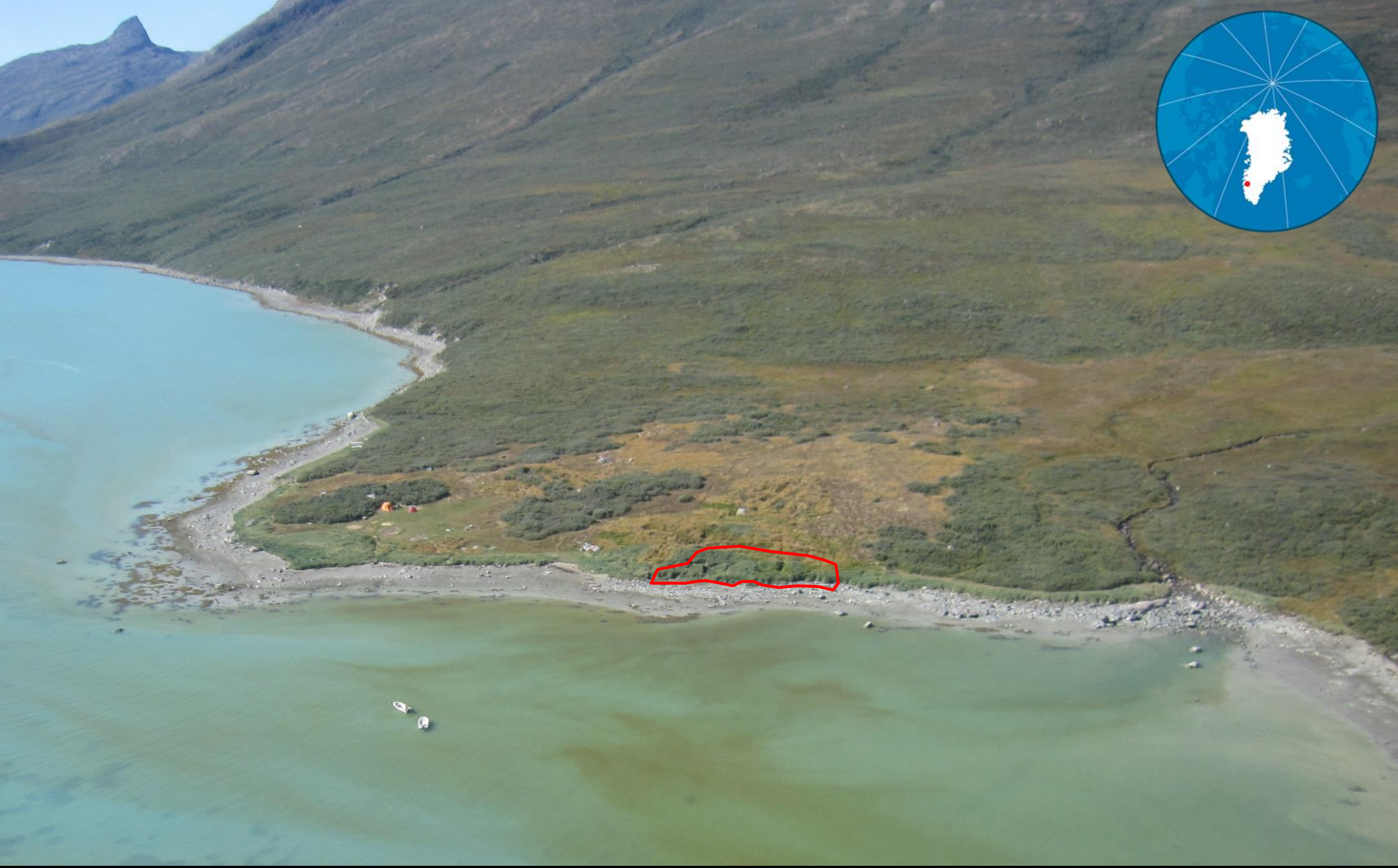

Supplementary figure S5: The midden at Sandnes (marked with red) is located between a Norse ruin and the shoreline of a small cape. (Photo: Jørgen Hollesen, National Museum of Denmark; Map: Kent Pørksen, University of Copenhagen).

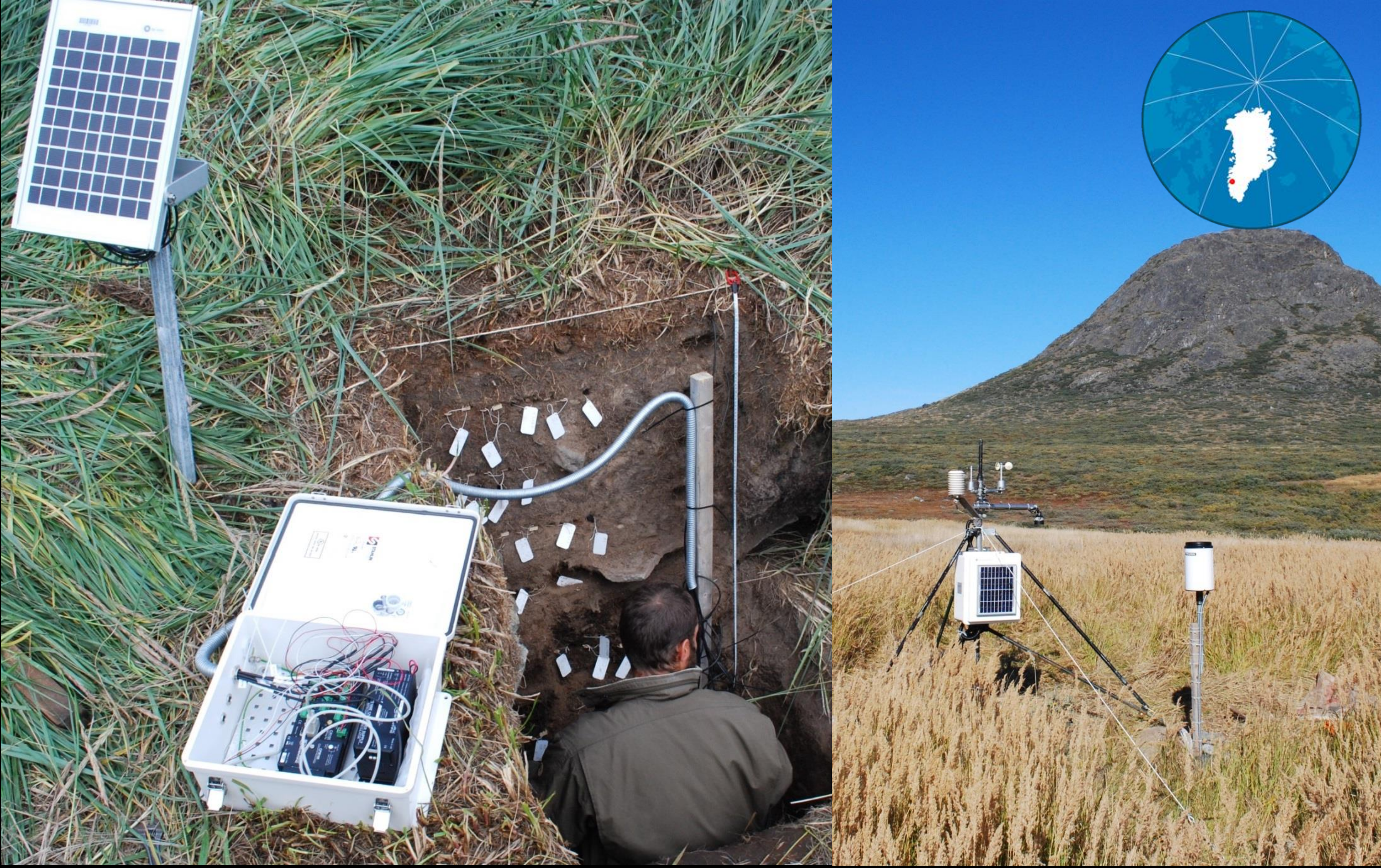

Supplementary figure S6: In 2012 a south-east facing profile from a previous excavation was cleaned at Sandnes. Samples were collected and temperature probes and soil water sensors were installed at various depths. Furthermore, a meteorological station was installed approximately 100 m west of the midden, 8 m.a.s.l (right). (Photo: Jørgen Hollesen, National Museum of Denmark; Map: Kent Pørksen, University of Copenhagen).

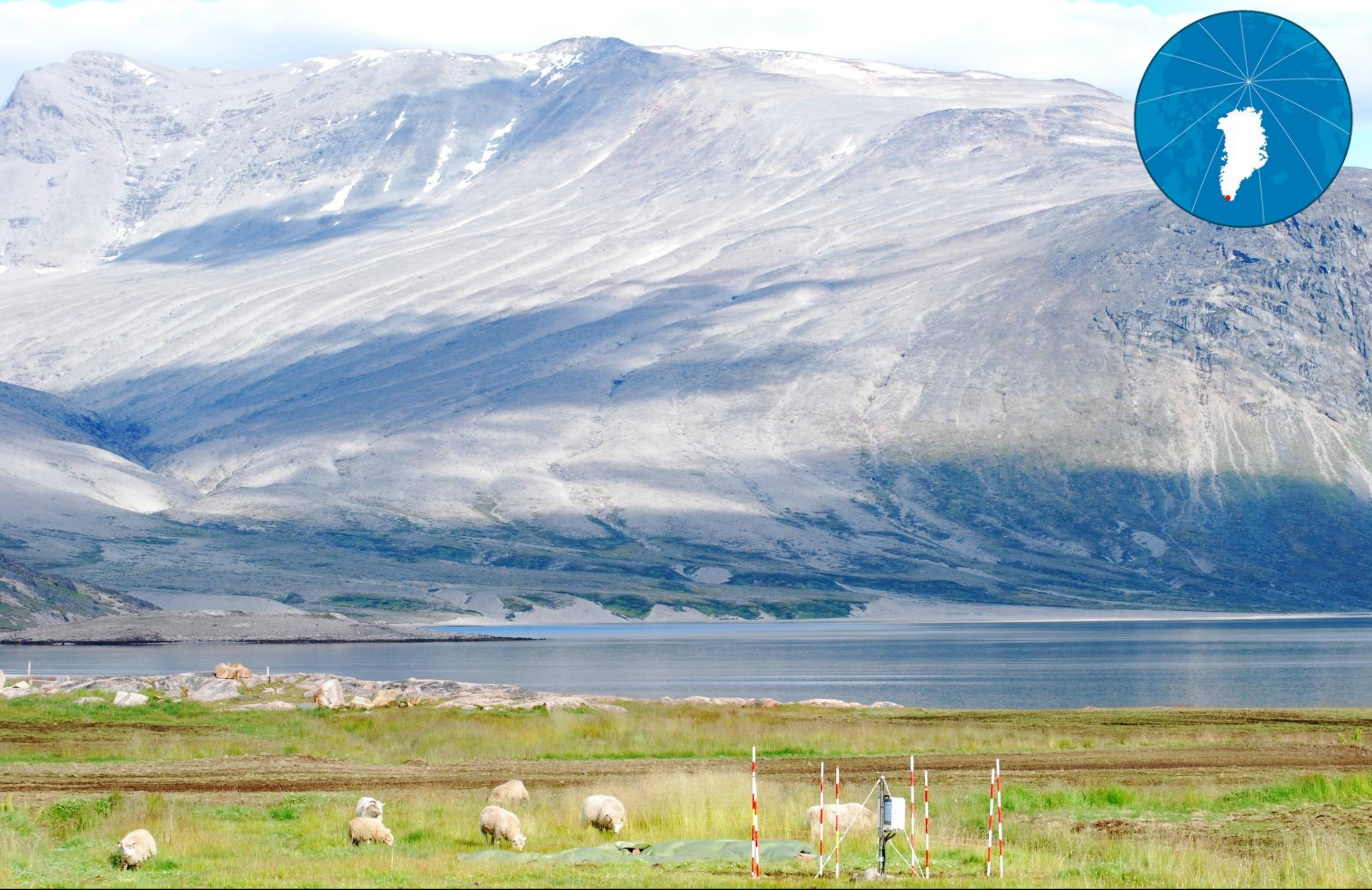

Supplementary figure S7: The cultural layers at Igaliku are located underneath the surface of a small wetland. This Norse site contains a large number of well-preserved artefacts including worked wood and animal bones. In 2012 two pits were excavated vertically into the cultural layers and temperature probes and soil water sensors were installed and connected to a datalogger. (Photo: Jørgen Hollesen, National Museum of Denmark; Map: Kent Pørksen, University of Copenhagen).

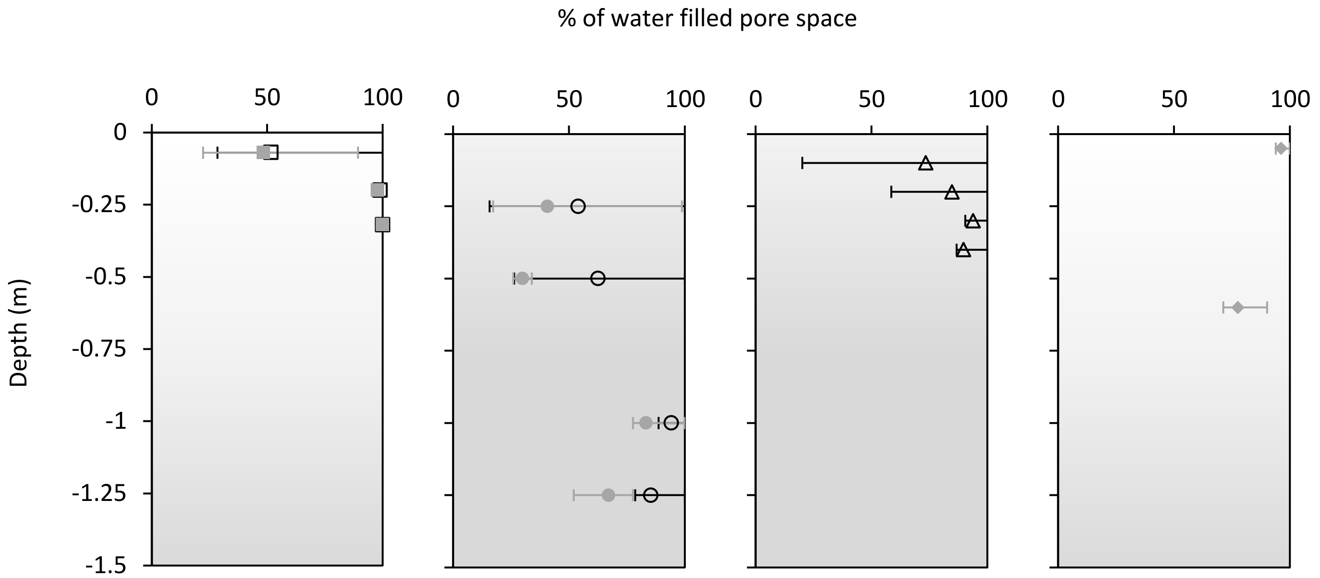

Supplementary figure S8 : Mean soil water content from 1 June to 31 August 2013 (open black symbols) and from 1 June to 15 August 2014 (filled grey symbols) at Qajaa (squares), Sandnes (circles), Kangeq (triangles) and Igaliku (diamonds). The horizontal bars show the variation from the minimum to the maximum daily means observed during the two periods.

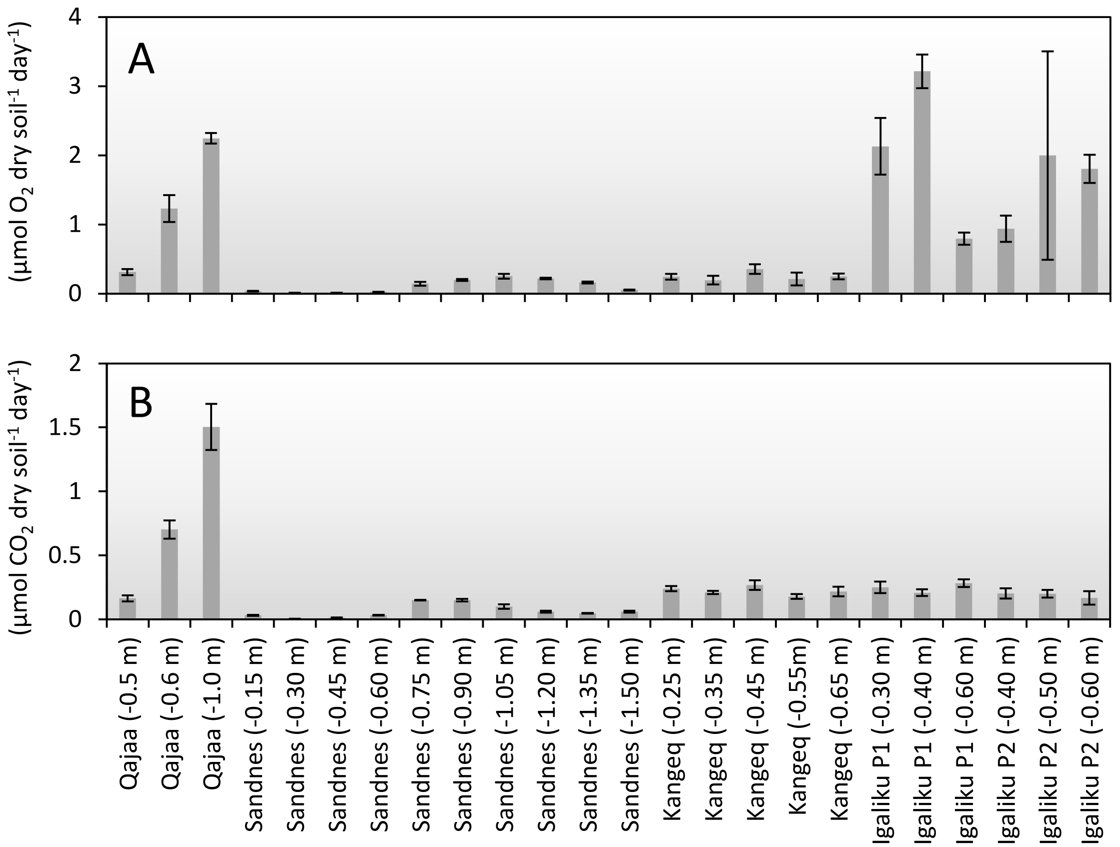

Supplementary figure S9: a) O_2 consumption and b) CO_2 production rates measured at 5 ^oC. Error bars show ±1 standard deviation (n =3).

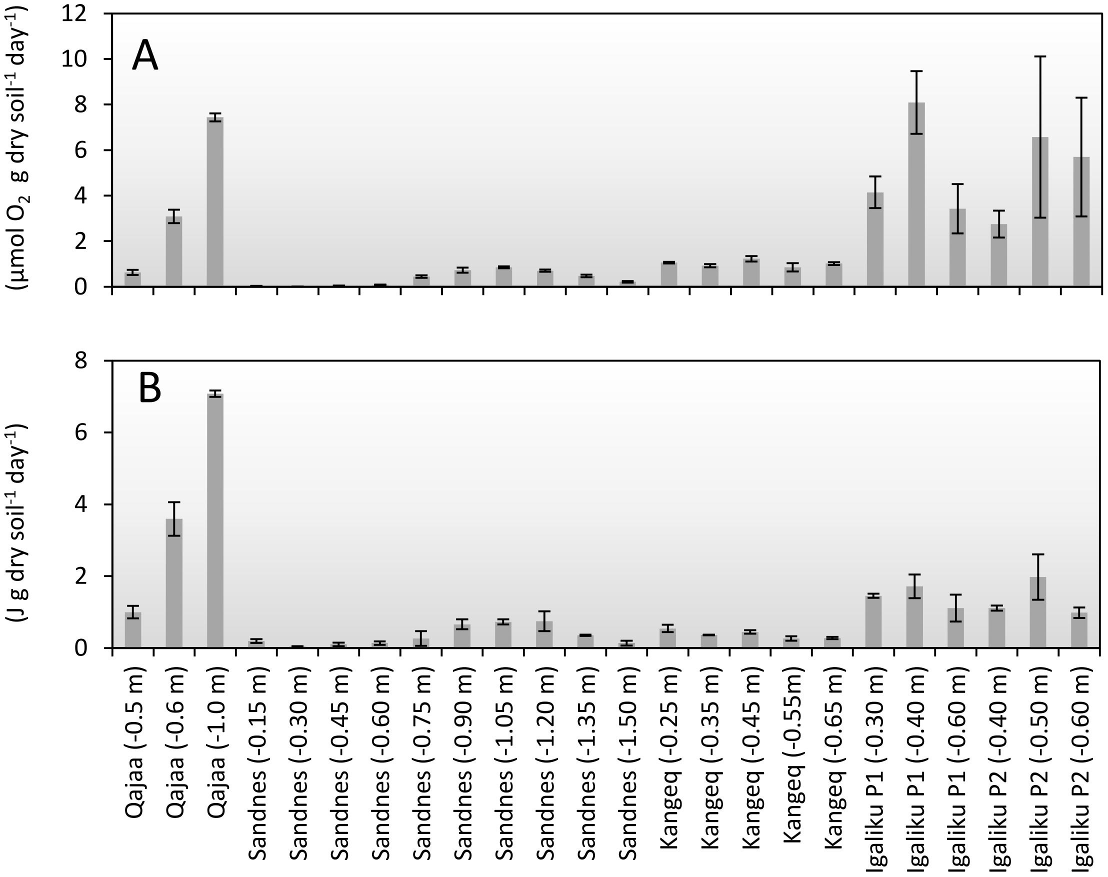

Supplementary figure S10: A) O_2 consumption and b) Heat production rates measured at 16 ^oC. Error bars show ± 1 standard deviation (n =3).

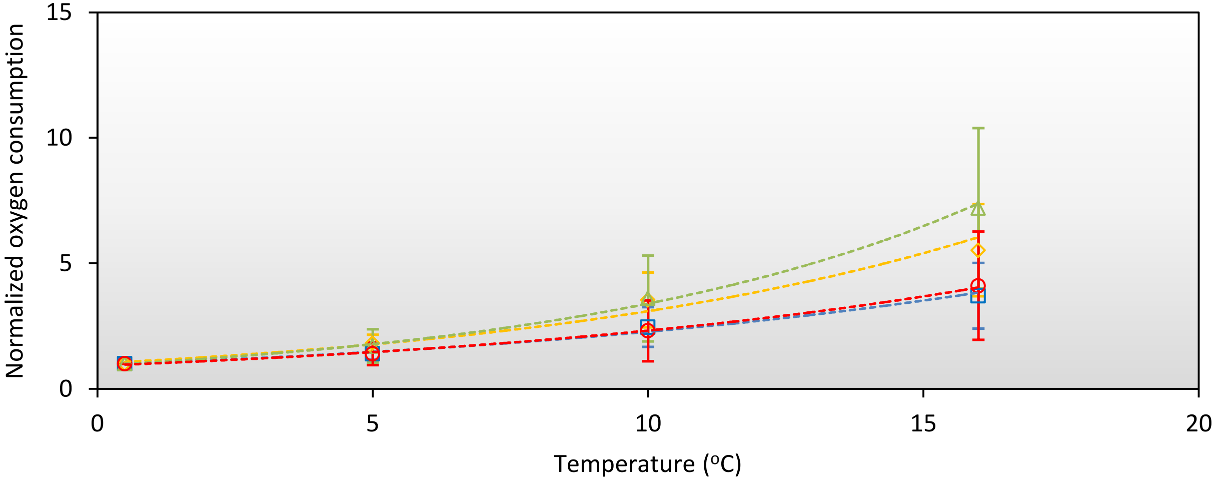

Supplementary Figure S11: O_2 consumption measured at 0.5, 5, 10, and 16 ^oC. Measurement were made on samples from Qajaa (Squares), Sandnes (circles), Kangeq (triangles), and Igaliku (diamonds). Error bars show ± 1 standard deviation (n =3). Data is normalized according to the minimum value. The Q_{10} values derived are shown in supplementary table S4.

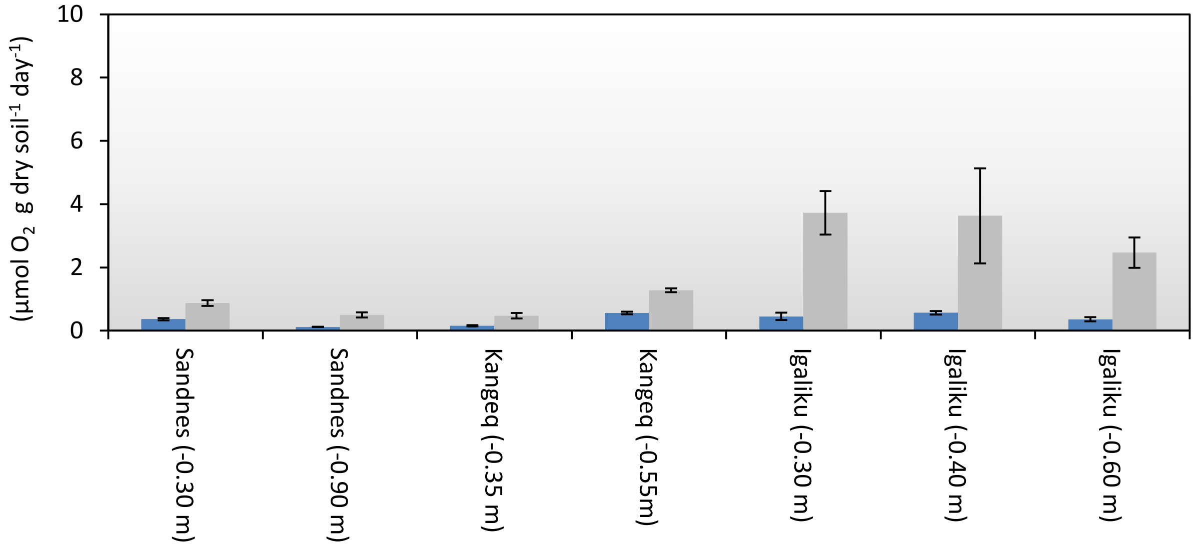

Supplementary Figure S12: O_2 consumption at saturated water contents (blue) and after 24 hours of draining (grey). Error bars show ± 1 standard deviation (n =3).

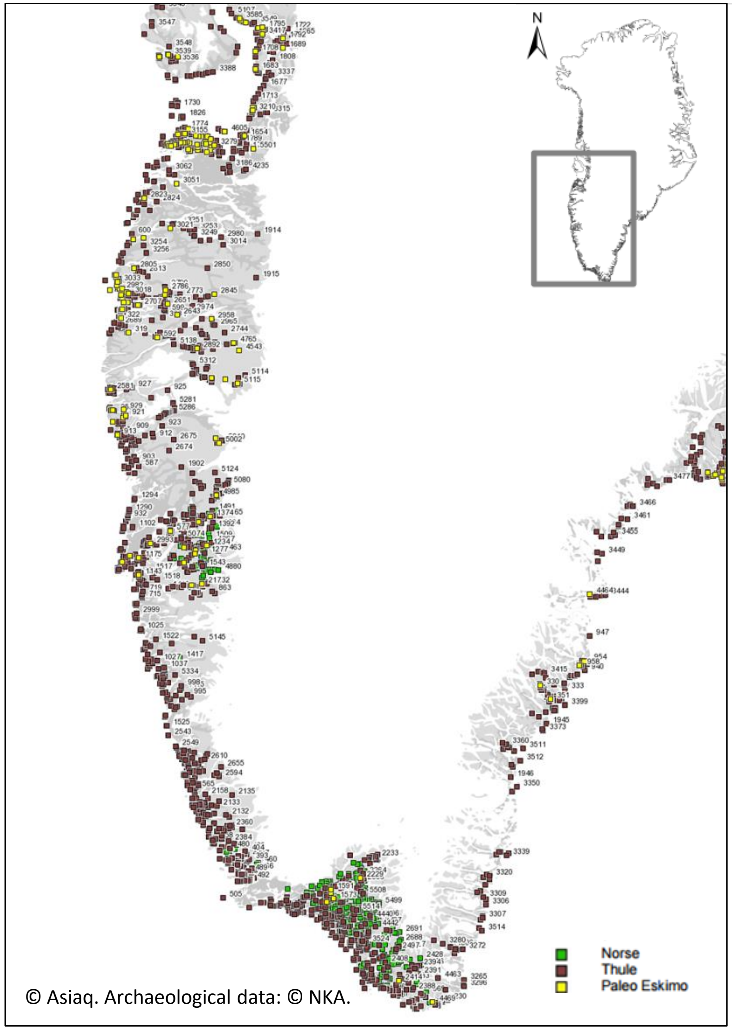

Supplementary Figure S13: Map of South and West Greenland featuring archaeological sites that are registered in the official Greenlandic heritage data base (Map: Greenlandic heritage data base, http://nunniffiit.natmus.gl/cbkort?).

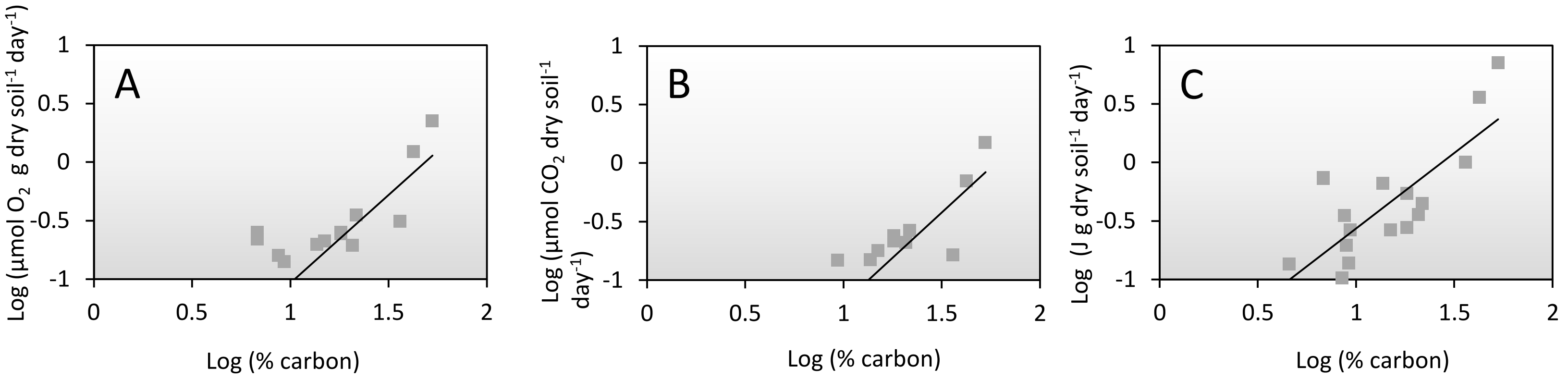

Supplementary Figure S14: Linear correlation between logarithmic transformed carbon content and A) O_2 consumption rates (5°C), B) CO_2 production rates (5°C), and B) Heat production rates (16°C), measured at Qajaa, Sandnes and Kangeq.

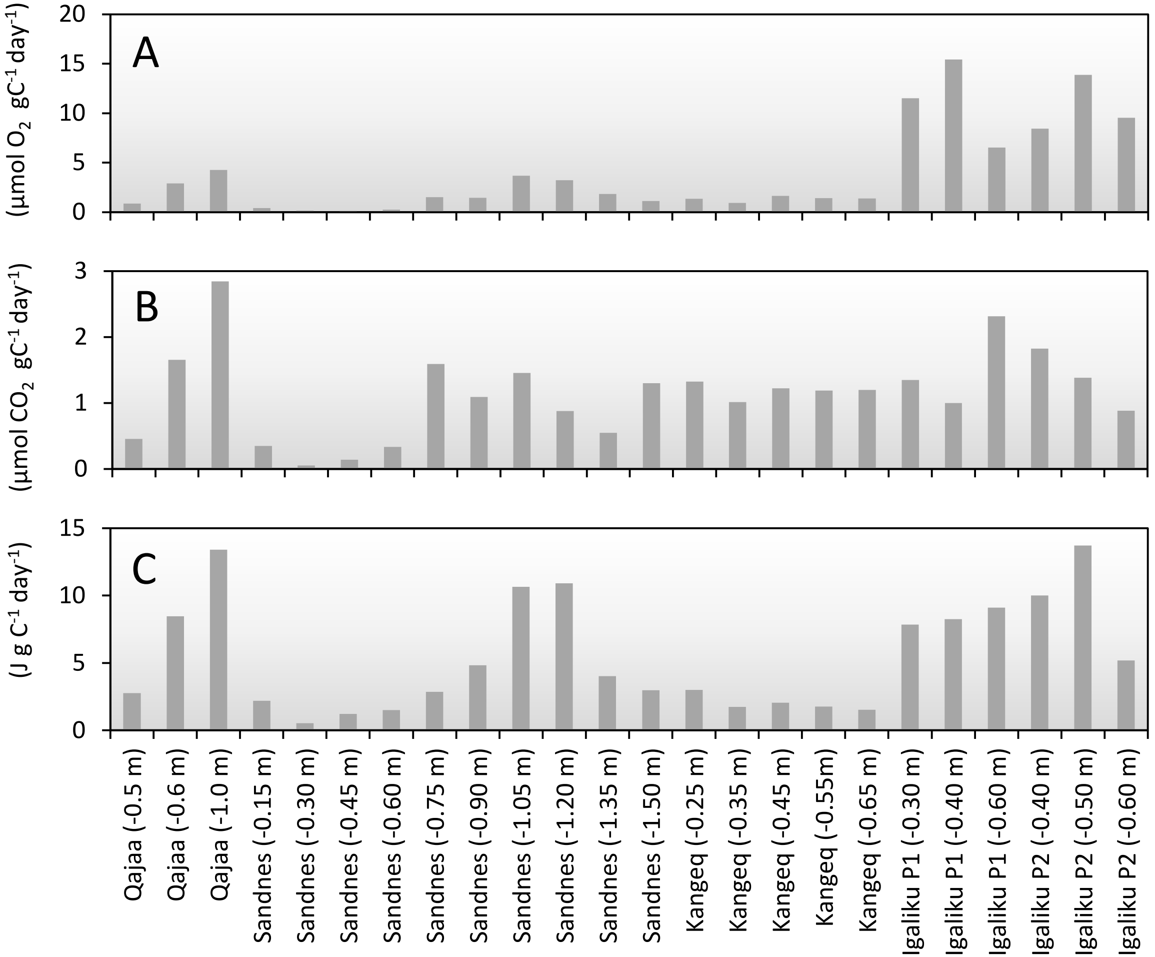

Supplementary Figure S15: A) O_2 consumption rates per g C (5°C), B) CO_2 consumption rates per g C (5°C), and C) heat production rates per g C (16°C).

Supplementary Table S1: Result of reclassification into 11 and 10 classes respectively with equal intervals for air temperature and precipitation. The total areas differ due to different data sets with different ground resolution, hence when masking unwanted surfaces out the number of affected pixels will differ.

**Estimated annual average air temperature**			**Estimated average annual precipitation**		
**Class**	Interval [^{o}C]	Area [km^2]	Class	Interval [mm year^{-1}]	Area [km^2]
**1**	-12.5 to -11	283	1	250 to 400	40414
**2**	-11 to -9.5	1466	2	400 to 550	16371
**3**	-9.5 to -8	4048	3	550 to 700	4610
**4**	-8 to -6.5	10092	4	700 to 850	16577
**5**	-6.5 to -5	22703	5	850 to 1000	13939
**6**	-5 to -3.5	34529	6	1000 to 1150	5832
**7**	-3.5 to -2	24963	7	1150 to 1300	3884
**8**	-2 to -0.5	15936	8	1300 to 1450	1174
**9**	-0.5 to 1	10309	9	1450 to 1600	1561
**10**	1 to 2.5	4955	10	1600 to 1750	218
**11**	2.5 to 4	290			
**Total**	-12.5 to 4	129574	Total	250 to 1750	104580

Supplementary Table S2: Spatial representativeness of the four study sites based on data from Supplementary Table 1. Numbers presented in parenthesis are based on areas < 100 meter above sea level only, where the majority of archeological sites in the area are found.

	Igaliku	Sandnes	Kangeq	Qajaa	Total representative area based on the reclassification
**Air temperature class**	8	9	9	6	51208 km^2<br>~40% of the analyzed area (~65% of the analyzed area).
**Precipitation class**	5	3	4	1	755400 km^2<br>~72% of the analyzed area

Supplementary Table S3: List of samples and the temperatures at which they were incubated for each type of measurement.

Site	Profile	Depth (m)	Incubation temperatures (°C)		
			O_2 consumption	Heat production	CO_2 production
**Qajaa**	A1	0.50	0.5, 5, 10, 16	16	5
**Qajaa**	A1	0.60	0.5, 5, 10, 16	16	5
**Qajaa**	A1	1.00	0.5, 5, 10, 16	16	5
**Kangeq**	Hole 6	0.25	0.5, 5, 10, 16	16	5
**Kangeq**	Hole 6	0.35	0.5, 5, 10, 16	16	5
**Kangeq**	Hole 6	0.45	0.5, 5, 10, 16	16	5
**Kangeq**	Hole 6	0.55	0.5, 5, 10, 16	16	5
**Kangeq**	Hole 6	0.65	0.5, 5, 10, 16	16	5
**Sandnes**	Profile 1	0.15	0.5, 5, 10, 16	16	5
**Sandnes**	Profile 1	0.30	0.5, 5, 10, 16	16	5
**Sandnes**	Profile 1	0.45	0.5, 5, 10, 16	16	5
**Sandnes**	Profile 1	0.60	0.5, 5, 10, 16	16	5
**Sandnes**	Profile 1	0.75	0.5, 5, 10, 16	16	5
**Sandnes**	Profile 1	0.90	0.5, 5, 10, 16	16	5
**Sandnes**	Profile 1	1.05	0.5, 5, 10, 16	16	5
**Sandnes**	Profile 1	1.20	0.5, 5, 10, 16	16	5
**Sandnes**	Profile 1	1.35	0.5, 5, 10, 16	16	5
**Sandnes**	Profile 1	1.50	0.5, 5, 10, 16	16	5
**Igaliku**	Profile 1	0.30	0.5, 5, 10, 16	16	5
**Igaliku**	Profile 1	0.40	0.5, 5, 10, 16	16	5
**Igaliku**	Profile 1	0.60	0.5, 5, 10, 16	16	5
**Igaliku**	Profile 2	0.40	0.5, 5, 10, 16	16	5
**Igaliku**	Profile 2	0.50	0.5, 5, 10, 16	16	5
**Igaliku**	Profile 2	0.60	0.5, 5, 10, 16	16	5

Supplementary Table S4: The Q_{10} values obtained from O_2 consumption measurements at 0.5, 5, 10 and 16 ^oC.

Site	Profile	Depth (m)	Q10 (0.5-16°C)	R (n=4)
**Qajaa**	A1	0.50	1.8	0.98
**Qajaa**	A1	0.60	2.6	1.00
**Qajaa**	A1	1.00	3.1	0.99
**Kangeq**	Hole 6	0.25	3.3	0.97
**Kangeq**	Hole 6	0.35	5.1	1.00
**Kangeq**	Hole 6	0.45	2.8	0.97
**Kangeq**	Hole 6	0.55	5.1	0.99
**Kangeq**	Hole 6	0.65	3.1	0.98
**Sandnes**	Profile 1	0.15	No result	
**Sandnes**	Profile 1	0.30	No result	
**Sandnes**	Profile 1	0.45	No result	
**Sandnes**	Profile 1	0.60	No result	
**Sandnes**	Profile 1	0.75	No result	
**Sandnes**	Profile 1	0.90	4.1	1.00
**Sandnes**	Profile 1	1.05	3.3	1.00
**Sandnes**	Profile 1	1.20	3.7	0.99
**Sandnes**	Profile 1	1.35	2.8	0.99
**Sandnes**	Profile 1	1.50	3.0	0.96
**Igaliku**	Profile 1	0.30	2.2	0.99
**Igaliku**	Profile 1	0.40	3.0	0.98
**Igaliku**	Profile 1	0.60	4.3	1.00
**Igaliku**	Profile 2	0.40	2.9	1.00
**Igaliku**	Profile 2	0.50	3.1	1.00
**Igaliku**	Profile 2	0.60	4.1	0.98
